# Cost effectiveness of the cancer prevention program for carriers of the *BRCA1/2* mutation

**DOI:** 10.11606/S1518-8787.2018052000643

**Published:** 2018-11-27

**Authors:** Marcelo Cristiano de Azevedo Ramos, Maria Aparecida Azevedo Koike Folgueira, Simone Maistro, Alessandro Gonçalves Campolina, Patricia Coelho de Soárez, Geertruida Hendrika de Bock, Hillegonda Maria Dutilh Novaes, Maria Del Pilar Estevez Diz

**Affiliations:** 1 Universidade de São Paulo. Faculdade de Medicina. Hospital das Clínicas. Instituto Central. Diretoria Executiva. São Paulo, SP, Brasil; 2 Universidade de São Paulo. Faculdade de Medicina. Hospital das Clínicas. Instituto do Câncer do Estado de São Paulo. Centro de Investigação Translacional em Oncologia. São Paulo, SP, Brasil; 3 Universidade de São Paulo. Faculdade de Medicina. Departamento de Medicina Preventiva. São Paulo, SP, Brasil; 4 University of Groningen. University Medical Center Groningen. Department of Epidemiology. Groningen, The Netherlands; 5 Universidade de São Paulo. Faculdade de Medicina. Hospital das Clínicas. Instituto do Câncer do Estado de São Paulo. Divisão de Oncologia Clínica. São Paulo, SP, Brasil

**Keywords:** Ovarian Neoplasms, diagnosis, Genes, *BRCA1*, Genes, *BRCA2*, Early Detection of Cancer, economics, Cost-Effectiveness Evaluation

## Abstract

**OBJECTIVE:**

To analyze the cost effectiveness of the diagnostic program for the germline mutation in *BRCA1/2* genes and of preventative strategies for the relatives of patients diagnosed with ovarian cancer associated with this mutation.

**METHODS:**

The study analyzed the cost effectiveness by developing an analysis of the Markov decision process from the perspective of the National Health System. The strategies compared reflect upon the adoption of genetic testing and preventative strategies for relatives or the usual care currently proposed. The incremental cost-effectiveness ratio was expressed in terms of cost per case avoided. The sensitivity analysis was performed in a univariate and deterministic manner.

**RESULTS:**

The study showed increments for effectiveness and for costs when performing genetic testing and adopting prophylactic measures for family members. The incremental cost-effectiveness ratio was estimated at R$908.58 per case of cancer avoided, a figure considered lower than the study's cost-effectiveness threshold (R$7,543.50).

**CONCLUSIONS:**

The program analyzed should be considered a cost-effective strategy for the national situation. Studies in various other countries have reached similar conclusions. One possible ramification of this research might the need to perform a budgetary-impact analysis of making the program one of the country's health policies.

## INTRODUCTION

According to data provided by the National Cancer Institute, around 6,150 new cases of ovarian cancer are recorded every year in Brazil^1^. Although this neoplasm only makes up a small proportion of the cases of malignancies diagnosed in the country, at the moment, it has the second highest mortality rate for all gynecological tumors^2^.

One of the main risk factors associated with the development of this neoplasm is genetic predisposition^3^ ^,^ ^4^. Hereditary breast and ovarian cancer syndrome, which is related to mutations in the *BRCA1* and *BRCA2* genes, is an important cause of ovarian carcinoma. It is present in 15% to 19% of all cases^5^ ^,^ ^6^. Germline *BRCA1/2* mutation testing is not currently funded by the National Health System, and the estimated prevalence rate of this mutation in Brazil is similar to several other studies.

Because of the strong association between ovarian carcinoma and germline mutation, different professional bodies have recommended counselling and genetic testing for any woman who develops a malignant tumor^7^ ^,^ ^8^. The benefit of testing is that the mutation significantly increases the risk of a second primary cancer and frequently affects the choice of treatment^9^. Performing a diagnostic exam also allows family members who carry the germline mutation to be identified, so they can be offered risk-reduction therapy^9^. The current recommendation to manage risk in women who are carriers of a mutation in the *BRCA1* and *BRCA2* genes consists of offering risk-reducing salpingo-oophoerectomy and bilateral prophylactic mastectomy^7^ ^,^ ^10^.

Therefore, the objective of this study was to analyze the cost effectiveness of the program for diagnosing the germline mutation in the *BRCA1/2* genes and of the preventative strategies offered to the relatives of patients who develop ovarian cancer.

## METHODS

### General Characteristics of the Study

The study analyzed the cost effectiveness of the program that diagnoses germline mutations in the *BRCA1/2* genes and of preventative strategies offered to the relatives of patients with ovarian cancer associated with this mutation. Germline mutation was already confirmed in these ovarian cancer patients, being either *BRCA1* or *BRCA2*.

**Figure 1:**
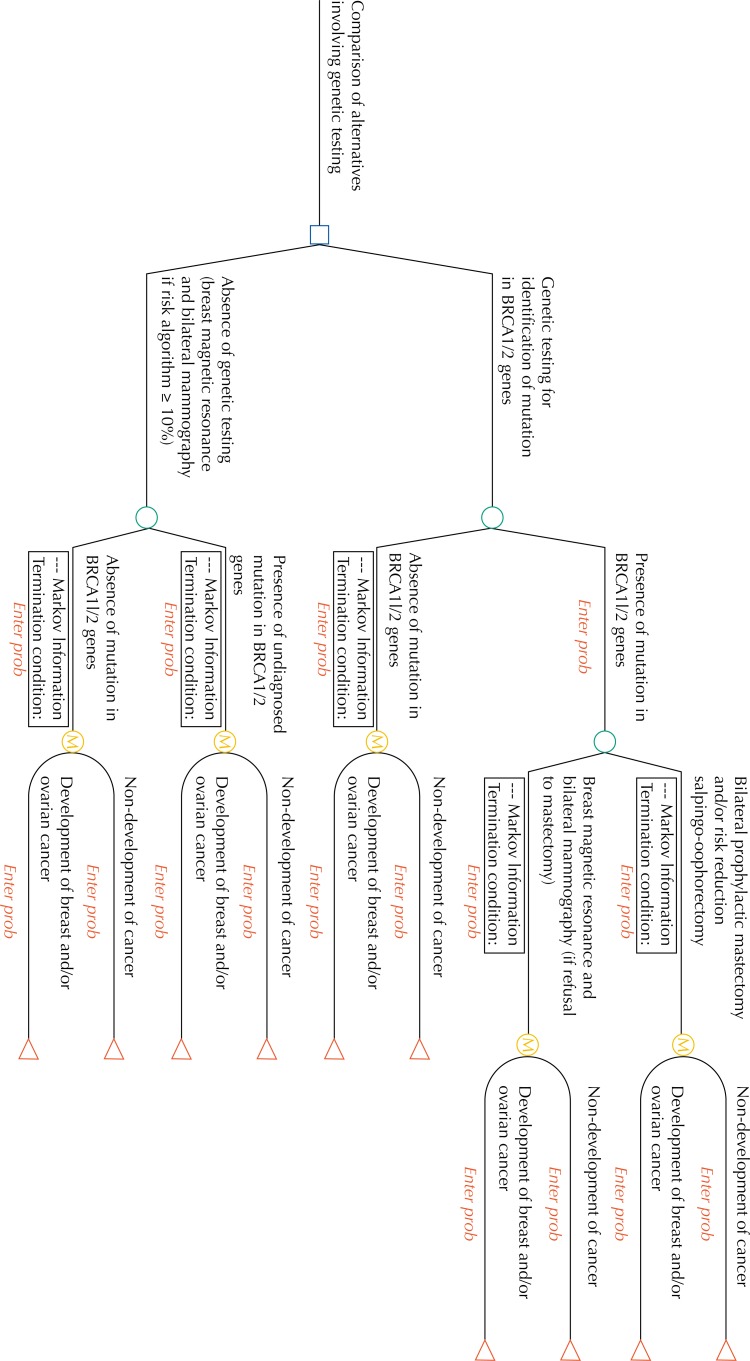
Markov model for strategies involving relatives of patients with ovarian cancer.

The perspective from the National Health System in the federal domain (Ministry of Health) was selected. The model of analysis for the decision adopted corresponded with the Markov model. This type of model was chosen because of the longer horizontal follow-up period and because of the need to simulate the transition between different states of health across fixed intervals of time. Figure 1 shows a consolidated version of the model proposed for this study.

The alternatives compared herein consisted of performing genetic tests on first-degree female family members of patients who have ovarian cancer and the germline mutation or the usual care currently proposed. In the group of family members who, hypothetically, underwent genetic testing, the simulation strategy consisted of offering risk-reducing salpingo-oophorectomy and bilateral prophylactic mastectomy when the germline mutation was present in the *BRCA1/2* genes. When mastectomy was refused, we simulated annual follow-up by complementary exams (magnetic resonance imaging of the breasts and a bilateral mammogram). The model did not adopt conducts aiming the early diagnosis of ovarian cancer, in situations where the salpingo-oophorectomy was refused, since the tracking methods currently available are not effective for this purpose^11^. No prophylactic conduct was simulated for family members without the germline mutation in the *BRCA1/2* genes. For each trajectory described, we simulated the possibility of subsequent development of malignant neoplasms of the breasts and ovaries.

In the group that, hypothetically, did not undergo genetic testing, annual follow-up with magnetic resonance imaging of the breasts and a bilateral mammogram were simulated when the algorithm of risk for the germline mutation was equal to or greater than 10%. No additional conduct was simulated when the algorithm of risk for mutation was lower than 10%. In addition, for each of the trajectories described, we represented the possibility of subsequent development of breast and ovarian cancers.

The model's temporal horizon extended from 30 to 70 years of age, with annual cycles to evaluate any transition in states and the development of cancer. Neither the age at which *BRCA1/2* testing was done nor the age at which risk-reducing salpingo-oophorectomy and bilateral prophylactic mastectomy were performed varied (30 years-old).

We adopted the following premises for this model: no family member had previously undergone genetic testing nor had a prior diagnosis of malignant breast and ovarian neoplasms, and family members who were not carriers of mutations in the *BRCA1/2* genes had the same risk as the general population for the development of malignant breast and ovarian neoplasms.

### Definition of the Estimates of Effectiveness

The definition of effectiveness was cancer cases prevented, discounted at 5%.

An umbrella research project provided demographic data, results related to the application of the algorithms of risk, and information about the prevalence of germline mutations in patients diagnosed with ovarian cancer in Brazil^6^ ^,^ ^12^.

Information related to the possible adherence to preventative strategies, penetrance of the germline mutations in the *BRCA1/2* genes, and the reduction of risk in the development of neoplasm by prophylactic surgery were obtained based on research in the PubMed database. In addition, we performed an electronic search for studies that did an economic evaluation of germline mutation in the *BRCA1* and *BRCA2* genes.

### Definition of the Estimates of Costs

The costs of genetic testing were defined based on commercial proposals received from manufacturers or local distributors. The monetary value in dollars or euros was converted into the Brazilian currency, based on the average quotations for the year 2014 (R$2.35 and R$3.12, respectively)^13^. The estimations involved measuring the costs for next-generation sequencing and for multiplex ligation-dependent probe amplification.

The sums involved in the program to reduce the risk of malignant neoplasm were defined by a panel composed of specialists and the hospital's clinical guidelines. The definition of costs involved the macro-costing method. The unit values corresponded to those described in the Table of Procedures, Medications and Ortheses, Prostheses, and Special Materials for the National Health System^14^. This reference point was chosen because it corresponds to the main component of health financing in Brazil, incorporating different geographic situations and health providers. Moreover, adopting a single pattern might help alleviate eventual distortions and discrepancies in the Table's values.

All data for the costs was presented in Brazilian Real for 2014. The annual discount rate for the costs adopted in the study was 5%, in line with the Guideline Methods for Economic Evaluation published by the Ministry of Health^15^.

### Demonstration of the Results and Sensitivity Analysis

Estimation and demonstration of the results of the economic evaluation were performed using the TreeAge Pro® sotfware, version 2017 (TreeAge Software Inc. Williamstown, Massachusetts). For each strategy compared, the anticipated costs and respective effectiveness were presented. The ratio of incremental cost effectiveness, estimated based on the mathematical ratio between the increment of costs and the increment of effectiveness, was expressed in terms of cost per case prevented. As a comparative reference, we used the cost-effectiveness thresholds developed by the Center for Health Economics at the University of York (R$7,543.50 to R$23,786.70)^16^.

We performed the sensitivity analysis in a deterministic and univariate manner. This analysis consisted of an individual evaluation of the main parameters of this study, while the others remained constant. The analysis was presented in graph form as a Tornado Diagram. The references for variability resulted in values for the 95% confidence interval for the parameters.

## RESULTS

The proportion of patients diagnosed with ovarian cancer and the germline mutation found in the project was 17% with the *BRCA1* gene and 2% with the *BRCA2* gene^6^. Given that the transmission of the mutation in autosomal dominant inheritance is 50%, the proportion of family members was estimated at 45% for carriers of the *BRCA1* gene mutation, 5% for carriers of the *BRCA2* gene mutation, and 50% for those without the mutation.

Penetrance of the mutations involving the *BRCA1* gene and breast cancer is more expressive than that for the *BRCA2* gene and ovarian cancer^17^. In fact, the probability of developing breast cancer is more prominent in the presence of a germline mutation in *BRCA1* (57%) than in *BRCA2* (49%)^17^. A similar situation occurs, though less often, for ovarian cancer, in which the probability of developing cancer is higher among carriers of a germline mutation in the *BRCA1* gene (40%) than among carriers of a germline mutation in the *BRCA2* gene (18%)^17^.

The parameters for adherence to risk-reducing surgeries and the impact of performing them on the future development of breast and ovarian cancer have also been determined^17^ ^–^ ^20^.

The costs involved next-generation sequencing and the evaluation of multiplex ligation-dependent probe amplification totaling R$683.61. The risk-reducing surgical procedures had the billing charges listed in the Table of Procedures, Medications, Ortheses, Prostheses and Special Materials of the National Health System.

The cost of screening for breast cancer in patients with a germline mutation in the *BRCA1/2* genes totaled R$333.75 per year. For family members who did not have genetic testing, annual expenditure was represented by the product of the probability of being at high risk for the mutation (24%) and the cost of screening (R$333.75), yielding a result of R$80.10.

Thus, based on the definition of the estimates of effectiveness and of the costs, we proceeded to develop Tables 1 and 2, which consolidate the parameters used in the model.

**Table 1:** Probabilities used in the decision model.

Description	Estimate	Range	Reference
Probability of mutation in *BRCA1* gene	0.45	-	Maistro et al.6 (2016)
Probability of mutation in *BRCA2* gene	0.05	-	Maistro et al.6 (2016)
Probability of adherence to bilateral prophylactic mastectomy	0.57	0.47-0.66	Chen et al.17 (2007)
Probability of adherence to risk reduction salpingo-oophorectomy	0.49	0.40-0.57	Chen et al.17 (2007)
Probability of developing breast cancer in the absence of mutation in *BRCA1*/2 genes	0.06	0.03-0.08	Ferlay et al.30 (2013)
Probability of developing ovarian cancer in *BRCA1* mutation carrier, in the absence of prophylactic surgery	0.40	0.35-0.46	Chen et al.17 (2007)
Probability of developing ovarian cancer in *BRCA2* mutation carrier, in the absence of prophylactic surgery	0.18	0.13-0.23	Chen et al.17 (2007)
Probability of developing ovarian cancer in the absence of mutation in *BRCA1/2* genes	0.006	0.005-0.01	Ferlay et al.30 (2013)
Probability of adherence to bilateral prophylactic mastectomy	0.18	0.16-0.20	Metcalfe et al.18 (2008)
Probability of adherence to risk reduction salpingo-oophorectomy	0.57	0.55-0.59	Metcalfe et al.18 (2008)
Probability of developing breast cancer in *BRCA1* mutation carrier, after mastectomy	0.04	0.03-0.05	De Felice et al.19 (2015); Chen et al.17 (2007)
Probability of developing breast cancer in *BRCA2* mutation carrier, after mastectomy	0.03	0.03-0.04	De Felice et al.19 (2015); Chen et al.17 (2007)
Probability of developing breast cancer in *BRCA1* mutation carrier, after salpingo-oophorectomy	0.28	0.23-0.32	Rebbeck et al.20 (2009); Chen et al.17 (2007)
Probability of developing breast cancer in *BRCA2* mutation carrier, after salpingo-oophorectomy	0.24	0.20-0.28	Rebbeck et al.20 (2009); Chen et al.17 (2007)
Probability of developing breast cancer in *BRCA1* mutation carrier, after mastectomy and salpingo-oophorectomy	0.02	0.01-0.03	De Felice et al.19 (2015); Rebbeck et al.20 (2009); Chen et al.17 (2007)
Probability of developing breast cancer in *BRCA2* mutation carrier, after mastectomy and salpingo-oophorectomy	0.02	0.01-0.02	De Felice et al.19 (2015); Rebbeck et al.20 (2009); Chen et al.17 (2007)
Probability of developing ovarian cancer in *BRCA1* mutation carrier, after salpingo-oophorectomy	0.08	0.07-0.10	Rebbeck et al.20 (2009); Chen et al.17 (2007)
Probability of developing ovarian cancer in *BRCA2* mutation carrier, after salpingo-oophorectomy	0.04	0.03-0.05	Rebbeck et al.20 (2009); Chen et al.17 (2007)

**Table 2:** Cost estimates used in the decision model.

Description	Cost
Genetic counseling consultations	R$200.00
Next generation sequencing and evaluation for large genomic rearrangements	R$683.61
Bilateral prophylactic mastectomy	R$3,158.04
Risk-reducing salpingo-oophorectomy	R$542.46
Breast magnetic resonance imaging and bilateral mammography, in the presence of BRCA1/2 germline mutation and refusal to mastectomy (annual cost)	R$333.75
Breast magnetic resonance imaging and bilateral mammography, in the presence of risk algorithm . 10% (annual cost)	R$80.10

The program to diagnose germline mutation and preventative strategies involving family members of patients with ovarian cancer proved to be cost effective, compared with the lowest threshold of cost effectiveness adopted (R$7,543.50). In fact, although the program had a higher cost (R$1,241.22 *versus* R$78.96), its effectiveness was better (R$16.13 *versus* R$14.85) than the strategy involving no genetic testing. Thus, the ratio of incremental cost effectiveness was estimated at R$908.59 per case of cancer prevented.

**Figure 2:**
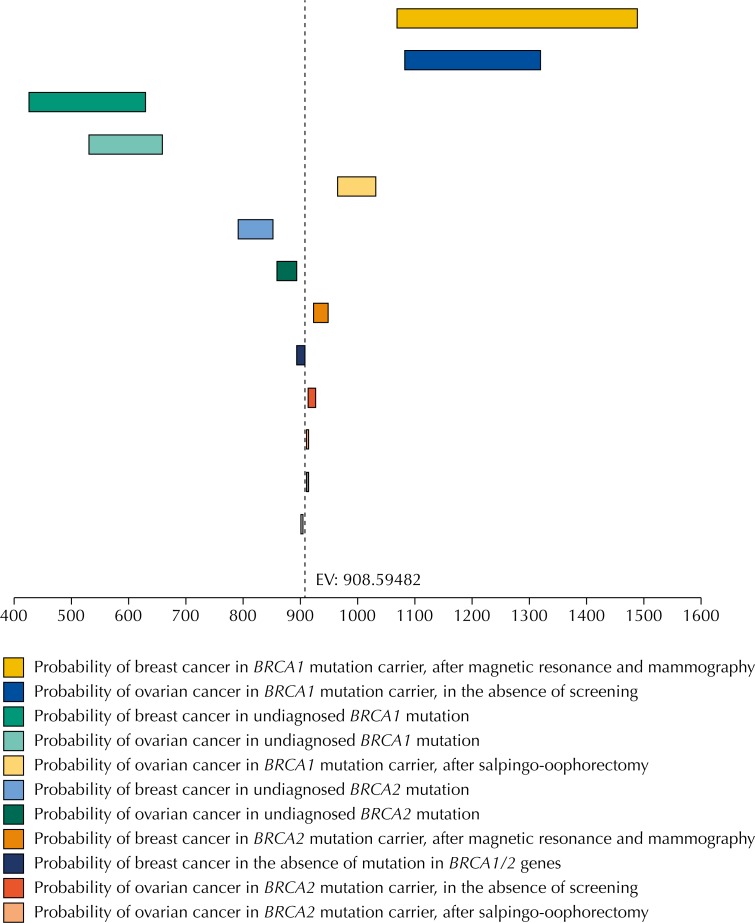
Tornado Diagram related to incremental cost-effectiveness ratio.

The Tornado Diagram showed that the parameters with the greatest influence on the model's results, during the univariate sensitivity analysis, were penetrance of the mutation in the *BRCA1* gene and the impact of not performing prophylactic surgeries. Individual variation in these parameters did not change the incremental cost-effectiveness ratio in an expressive way, so that the technology remained cost effective. Figure 2 illustrates this situation.

Finally, the number of family members eligible for the strategy, per year, was estimated at 2,045 individuals (6,150 cases of ovarian cancer per year multiplied by 19% of these cases related to *BRCA1/2* germline mutation multiplied by 3.5 first-degree female relatives per patient with ovarian cancer, multiplied by 50% probability of transmission of the mutation).

## DISCUSSION

The program for diagnosing a germline mutation in the *BRCA1/2* genes and for preventative strategies, aimed at family members of patients diagnosed with ovarian cancer, proved to be a cost-effective alternative, from the perspective of the National Health System. It is a strategy located in quadrant I of the cost-effectiveness plan, consisting of a more effective and more costly intervention^21^. In this case, the comparison between the incremental cost-effectiveness ratio and the cost-effectiveness threshold proved favorable to the adoption of the program.

In addition, the cost per case of cancer prevented (R$908.59) was lower than the amounts typically spent on treating the cancer. In fact, data from our cohort of patients with ovarian cancer had more expressive billing figures (Table 3), over a period of five years of tracking, with palliative chemotherapy as the main driver of costs. In this respect, adopting prophylactic strategies for carriers of a germline mutation in the *BRCA1/2* genes could reduce costs and need for palliation in cases of tumor progression.

**Table 3:** Costs of ovarian cancer treatment by year.

Year	Mean cost	95%CI
1st	R$12,958.48	R$11,504.42-R$14,412.54
2nd	R$3,960.10	R$2,783.99-R$5,136.22
3rd	R$4,860.90	R$3,597.02-R$6,124.78
4th	R$4,167.97	R$2,934.69-R$5,401.24
5th	R$3,508.02	R$1,993.53-R$5,022.51

The indication of genetic testing for patients with ovarian cancer and the subsequent adoption of preventative strategies for family members carrying the germline mutation was also the motive behind a North American study^22^. This study concluded that testing all cases of ovarian cancer could be prohibitively expensive in comparison with the cost-effectiveness threshold adopted in the United States^22^. On the other hand, restricting the test to situations of prior personal history of breast cancer, family history of malignant breast and ovarian neoplasms or Ashkenazi Jewish background showed cost-effective results^22^.

In this context, different studies emphasize evaluation, at the level of population, of preventative strategies for people with a higher risk of having a germline mutation^23^ ^–^ ^26^ One of these publications, analyzing conduct for individuals with a predictive algorithm of risk for the *BRCA1/2* mutation equal to or higher than 10%, proved inconclusive regarding the use of technology, since the costs and effects of the alternatives adopted are similar to not having genetic testing^23^.

Three different publications analyzed a specific group at high risk for the germline mutation in the *BRCA1/2* genes: Ashkenazi Jews^24^ ^–^ ^26^. The studies assessed specific sequencing for founder mutations. Thus, Rubinstein et al.^25^ concluded that the screening program for the germline mutation increased survival at an acceptable cost. One limitation of this study, which focused entirely on ovarian cancer, was the lack of strategies to reduce the risk of breast cancer^25^. Manchanda et al.^24^, in turn, verified that a population-based screening strategy could save money and promote greater effectiveness by performing genetic tests only on people with a family history of malignant neoplasm. Finally, Grann et al.^26^ showed the superiority of combined surgery over exclusive mastectomy, exclusive salpingo-oophorectomy and the usual practice of watching and waiting for this population.

Other studies have assessed preventative strategies for individuals who carry the germline mutation in the *BRCA1* and *BRCA2* genes^27^ ^–^ ^29^. Thus, the study conducted by Anderson et al.^29^ showed a favorable incremental cost-effectiveness for the combination of bilateral prophylactic mastectomy and risk-reducing salpingo-oophorectomy. Another publication, which assessed preventative strategies for carriers of a mutation in the *BRCA1* gene, reached a similar conclusion, pointing out that salpingo-oophorectomy, with or without bilateral prophylactic mastectomy, should be cost effective^28^. Similarly, Grann et al.^27^ suggested that, for carriers of a germline mutation in the *BRCA1/2* genes, prophylactic surgery is cost effective in comparison with chemoprevention and screening.

Therefore, the overall verdict of this study is that the program for diagnosing a germline mutation in the *BRCA1/2* genes and for preventative strategies should be considered cost effective when its impact on family members of patients diagnosed with ovarian cancer is assessed. Different studies published in the literature have also shown the cost effectiveness of performing genetic tests and of risk-reducing surgical procedures.

One potential limitation of this study is the use of estimates and probabilities from international observational studies, since national data was unavailable. The use of billing information may also have caused distortions because of potential discrepancies regarding the cost of procedures. However, this perspective has the advantage of representing the actual payments made by the National Health Service in the federal domain.

Finally, the determination of the incremental cost-effectiveness ratio, even though it is very useful for informed decision-making, is not enough for the incorporation of technologies into the National Health System^15^. That decision can be influenced by many variables, such as political interest and priorities, social preferences, concerns about equity, financial availability, and budgetary impact. Thus, one possible ramification of this study would be the need to analyze the budgetary impact of turning the program into one of the country's health policies. Although the technology is considered cost effective, it is essential to know how much money the program would cost if it were offered by the National Health System to the Brazilian population. Such an analysis could guide decision-making and public policies for conditions such as ovarian cancer, which are highly lethal and often diagnosed quite late.
